# The comparison of four mitochondrial genomes reveals cytoplasmic male sterility candidate genes in cotton

**DOI:** 10.1186/s12864-018-5122-y

**Published:** 2018-10-26

**Authors:** Shuangshuang Li, Zhiwen Chen, Nan Zhao, Yumei Wang, Hushuai Nie, Jinping Hua

**Affiliations:** 1 Laboratory of Cotton Genetics, Genomics and Breeding/Key Laboratory of Crop Heterosis and Utilization of Ministry of Education/Beijing Key Laboratory of Crop Genetic Improvement, College of Agronomy and Biotechnology, China Agricultural University, Beijing, 100193 China; 2 Institute of Cash Crops, Hubei Academy of Agricultural Sciences, Wuhan, 430064 Hubei China

**Keywords:** Mitochondrial genomes, Cytoplasmic male sterility, Chimeric ORFs, Comparative genomics, Transcriptomes, *Gossypium*

## Abstract

**Background:**

The mitochondrial genomes of higher plants vary remarkably in size, structure and sequence content, as demonstrated by the accumulation and activity of repetitive DNA sequences. Incompatibility between mitochondrial genome and nuclear genome leads to non-functional male reproductive organs and results in cytoplasmic male sterility (CMS). CMS has been used to produce F_1_ hybrid seeds in a variety of plant species.

**Results:**

Here we compared the mitochondrial genomes (mitogenomes) of *Gossypium hirsutum* sterile male lines CMS-2074A and CMS-2074S, as well as their restorer and maintainer lines. First, we noticed the mitogenome organization and sequences were conserved in these lines. Second, we discovered the mitogenomes of 2074A and 2074S underwent large-scale substitutions and rearrangements. Actually, there were five and six unique chimeric open reading frames (ORFs) in 2074A and 2074S, respectively, which were derived from the recombination between unique repetitive sequences and nearby functional genes. Third, we found out four chimeric ORFs that were differentially transcribed in sterile line (2074A) and fertile-restored line.

**Conclusions:**

These four novel and recombinant ORFs are potential candidates that confer CMS character in 2074A. In addition, our observations suggest that CMS in cotton is associated with the accelerated rates of rearrangement, and that novel expression products are derived from recombinant ORFs.

**Electronic supplementary material:**

The online version of this article (10.1186/s12864-018-5122-y) contains supplementary material, which is available to authorized users.

## Background

Cytoplasmic male sterility (CMS), a phenomenon that the male reproductive structures fail to develop, is an important agronomical trait in higher plants. The CMS character is frequently used in crop breeding and commercial seed production to increase the yield of the crops such as rice, maize, rapeseed, and cotton [1–5]. CMS is maintained by the maintainer line which is similar to the CMS line in terms of the nuclear composition but is equipped with the male-fertile cytoplasm. Fertility is conferred by a third line which carries the nuclear restorer genes [6–8]. The CMS phenotype is closely associated with mutations in mitochondrial genomes (mitogenomes) [9, 10]. The different CMS phenotypes are the results of frequent recombination, gene shuffling and mutation [11, 12]. In CMS lines, mitogenomic sequences’ rearrangements produce chimeric genes which disrupt the normal physiological functions and cause male gametophyte abnormalities, such as pollen abortion [13]. Novel chimeric genes responsible for CMS were identified by evaluating the difference in mtDNA and transcriptional products among the following lines: CMS, maintainer, and restorer in maize [14], wheat [15], rice [16], pepper [17] and rapeseed [18–20]. Unfortunately, there are few reports on mitochondrial genomes rearrangement and the role of CMS in cotton.

Next-generation sequencing technology (NGS) has been applied to plant chloroplast (cp) genomes, with over 1200 species sequenced [21–25]. However, plant mtDNA has a large number of repeats sequences and rearrangements, thus limiting the use of NGS [26–32]. Likewise, RNA sequencing has been broadly used to study plant transcriptome and mtDNA [33–37]. However, the focuses of most studies were on mitochondrial global transcript levels. Comparative analysis of the mitochondrial transcriptomes of CMS, maintainer, F_1_ and restorer lines’ in the context of their nuclear genomes can provide insights into cytonuclear-related phenotypes, such as cytoplasmic male sterile [11, 38]. In this study, we performed a comparative analysis of the mtDNA of the CMS, restorer and maintainer lines from both *Gossypium harknessii* and *G. hirsutum* to determine candidate CMS factors. We also analyzed the expression patterns of uncharacterized ORFs, some of which are candidate genes for CMS. The results give some interesting clues about mitochondrial evolution and CMS generation, as well as generate a background for future studies on CMS molecular diversity and phenotypic variability in cotton.

## Methods

### Materials and mtDNA preparation

Line 2074A, an upland cotton cytoplasmic male sterile line with *Gossypium harknessii* Brandegee CMS-D_2–2_ cytoplasm, was from its original sterile line DES-HAMS277. Line 2074S, an upland cotton cytoplasmic male sterile line, was from *G. hirsutum* L. CMS-AD_1_. These two lines were genetically stable cotton sterile lines derived from 20 to 30 generations of backcross. Line 2074B, a cultivar of upland cotton ‘Sumian No. 20’, was the maintainer of these two cytoplasmic male sterile lines. The restorer E5903 is a nuclear restoring line with normal nuclear and normal male-fertile *Gossypium harknessii* Brandegee. 2074A, 2074S and E5903 cotton materials used in this study are developed in our own lab [39]. We breed these three cotton cultivars and the work started 20 years before. The mtDNA preparation was performed described previously [39].

### Library construction and genome sequencing, assembly and sequence verification

The mitogenome Fosmid library was constructed according to the manufacturer’s protocols (MaxxPlaxTMLambda Packing Extract)/(CopyControlTM Fosmid Library Production Kits; Epicentre Technologies, Madison, WI). All these three mtDNAs Fosimd libraries have been constructed and screened with probes from sequences of conservative genes and scaffolds. From those libraries, 1000 clones were randomly selected and screened with 28 probes designed from sequences of mitochondrial genes. At last, 22, 26 and 21 positive clones were obtained from 2074A, E5903 and 2074S, from which 23 clones (seven for 2074A, nine for E5903, and seven for 2074S) were selected to cover larger repeats and sequenced the double-ends by shotgun strategy, with insert size of about 36.2 kb- 38.4 kb. Sequenced fragments were aligned using Blastn to determine the exactness of assembly [39].

The mtDNA samples were sequenced using Illumina strategy at BGI (Beijing Genomics Institute) and assembled primarily using SOAPdenovo [40]. The Illumina system produced 413–607 M usable reads in one run for genome assembly and about 700 × coverage with Solexa using paired-end (90 bp reads). Raw sequences were evaluated by two quality control tools, using the Trimmomatic [41] and FilterReads module in Kmernator (https://github.com/JGI-Bioinformatics/Kmernator) to remove potential undesirable artifacts, including adapters or low-quality or N bases or short sequences. The filtered reads Q30 > 85%. These filtered reads were a mixture of reads derived from chloroplast, mitochondrial and nuclear genomes; firstly, we removed the chloroplast and nuclear contaminant contigs through Blastn against nt/nr database (Additional file 1: Table S1). Through adjusting the software SOAPdenovo with the reasonable parameters (−s config_file -K 37 -R -D 1 [40]), we acquired 28–65 big contigs in 4 mitogenomes. Among them, the mitogenomic sequences of 2074B had been published and the sequence was deposited in GenBank database under the accession number: JX065074.1 [42]. In addition, known mitogenomic sequences from our previous studies, including *G. hirsutum* 2074B [42], *G. barbadense* [43], *G. raimondii* and *G. arboreum* [30], as well as eight other diploid and tetraploid species [31], were used to order/orient mitochondrial-type scaffolds.

Combined with the scaffolds’ information and one whole-genome backbone with positive clones, three procedures were adopted to finish the physics gaps. Firstly, we screened the library of the whole mitogenome according to the splicing sequence and the functional genes, constructed genome physical map and then sequenced the positive clones [39]. Secondly, according to the relationship of whole-genome physics map with the positive clones, we designed primers combination on the different scaffolds’ terminals, and used long-PCR to finish the gaps (Additional file 2: Table S2A). Thirdly, PCR amplification was performed based on primers pairs that consist of the terminal sequences of large repeats (Additional file 3: Table S2B). Finally, we assembled three circle mitogenomes (2074A, 2074S, E5903). To evaluate the quality and accuracy of these three mitogenomic sequences’ assemblies, pair-end reads were mapped onto their respective consensus sequences with BWA 0.7.10-r789 [44]. The resulting SAM files from BWA mapping were transformed into BAM files using samtools view program [45]. The BWA mapping results of these pair-end reads in BAM files were then used to calculate the depth of sequencing coverage using samtools depth program [45]. For three *Gossypium* species, the Illumina reads covered all parts of the genomes consistently, achieving an average sequencing depth of 214.3× in 2074A mitogenome (clean data, 413 M), 28.8× in 2074S mitogenome, 27.3× in E5903 mitogenome.

### Analyses and annotations of mitogenomes and sequence data

Intersubspecific polymorphisms were firstly identified based on the MUMmer package (v3.06) [46]. The results were acquired using a custom-designed Perl script and were confirmed through careful visual inspection. We carried out analyses on repeat sequences using the Washington University (WU)-Blast, including forward, palindromic reverse, and complemented repeats with a minimal length of 20 bp. Cp-derived (chloroplast-derived) sequences were identified using BlastN search of mitogenomes against annotated cotton chloroplast genomes (Identity ≧90%, E-value ≦1e-5, and Length ≧30 bp). Nuclear-derived insertions were searched against the *G. raimondii* genome. The syntenic regions of mitogenomes between different cultivars were detected using Nucmer of the MUMmer package (v3.06) [47] with 50 bp exact minimal match. NCBI-BlastX and -BlastN searches of the genomes against databases of sequenced plant mitogenomes were performed to find protein-coding and structural RNA genes, respectively. tRNA genes were searched by tRNAscan-SE [48] and were identified by BlastN [47]. The annotated mitogenomes features, including gene coordinate and genome structures, in genomes were drawn by OGDRAW v1.1 [49] and R Project (https://www.R-project.org/).

We used YASS to analyze the genome complexity that was defined as the complete sequence information of a genome with only one copy of each duplicate (> 500 bp). We set parameters as follows: E-value < 1e-30 (with the score “+ 1” for one match and “-3” for one substitution); the rate of substitutions and insertions/deletions < 5% [50].

### Analysis of candidate cytoplasmic male sterility genes

Based on the previous reports showing that CMS genes are chimeric [3, 7, 51, 52], a search for chimeric ORFs was conducted. Open reading frames (ORFs) were identified by ORFfinder (https://www.ncbi.nlm.nih.gov/orffinder/) and EMBOSS (6.3.1: getorf) [53]. All ORFs at least 300 bp in length were compared to the mitogenomes of the maintainer line 2074B and the restorer line E5903 using BlastN with an identity of 99% and an E-value cut off of 1 × 10^− 5^. ORFs containing at least 30 bp of an identified mitochondrial gene were characterized as chimeric, excluding any ORFs that overlap the genomic position of an identified gene. Transmembrane domains in each candidate ORF were predicted using TMHMM Server version 2.0 (http://www.cbs.dtu.dk/services/TMHMM/).

### Sequencing of the cotton mitochondrial transcriptome

The extracted mitochondrial RNA from the flower buds (3–5 mm size) in CMS line 2074A, its maintainer 2074B and fertile material F_1_ (2074A × E5903) were sequenced on an Illumina HiSeq2000 at BGI (Beijing Genomics Institute). Ribosomal RNAs were removed from the extracted mitochondrial RNA using Ribo-Zero (Epicentre, Madison, WI) and the mitochondrial RNA libraries were prepared using Illumina’s TruSeq RNAseq Sample Prep kit. Libraries were sequenced on one lane with 4 Gb clean reads/samples of an average length of 90 nt for paired-end. RNA sequence data quality was checked using FastQC to remove the adapters, low-quality, containing N bases and short sequences with reads Q30 > 85%. The reads were mapped to the assembled mitogenome of CMS line 2074A using bowtie2 [54] with the following parameters: -D 5 -R 1 -N 0 -L 25 -i S, 1, 2.00. Then, the resulting SAM files from bowtie2 mapping were transformed into BAM files using samtools view program [45]. The bowtie2 mapping results of these pair-end reads in BAM files were then used to calculate read count for each gene through HTSeq-count program [55]. Differentially expressed genes that showed up and down regulation between samples were defined based on the standards of cutoff: two-fold change and a *p*-value of less than 0.05.

## Results and discussion

### Structures and contents of CMS, maintainer, and restorer mitochondrial genomes

**Fig. 1:**
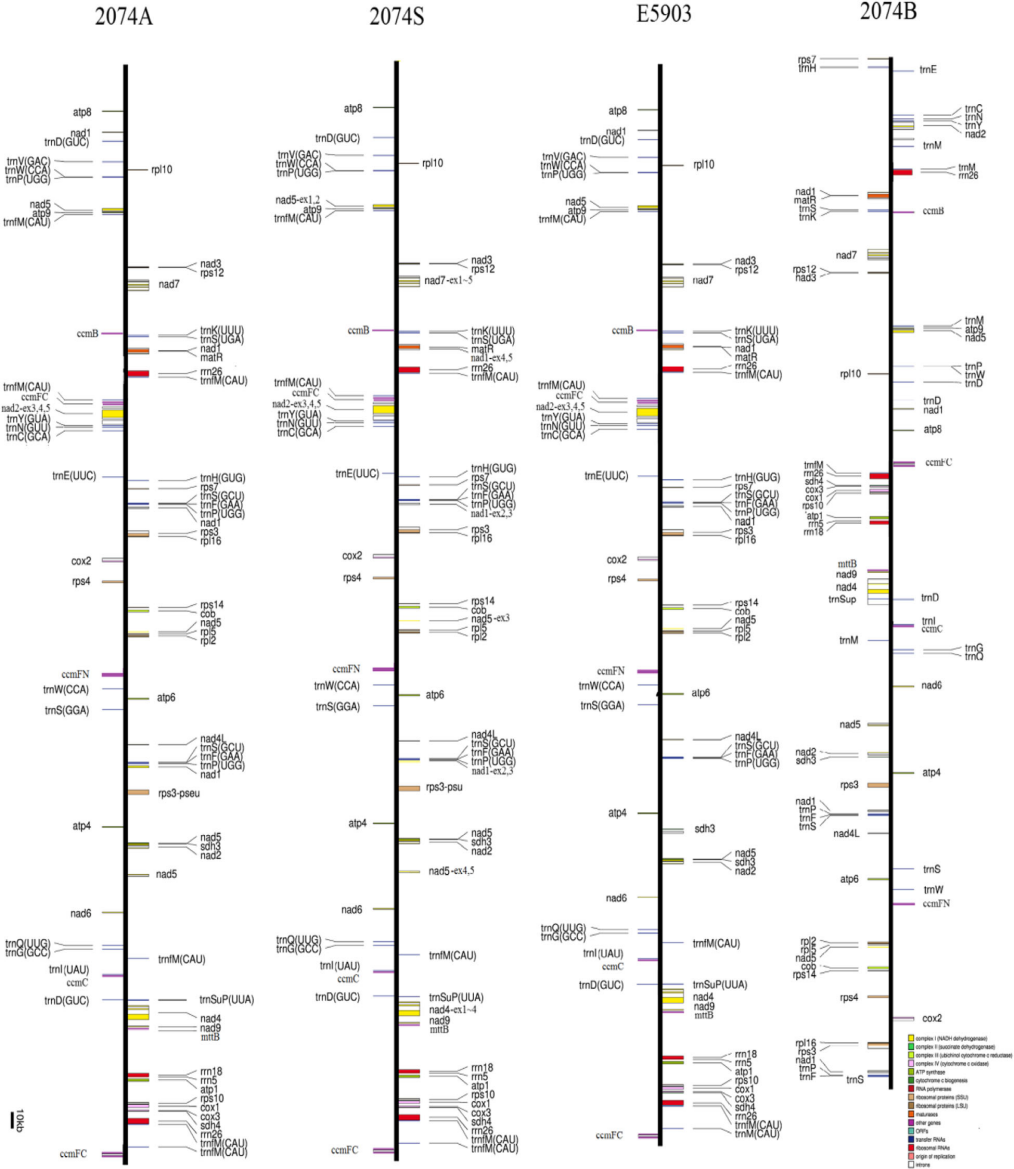
Linear maps of the four cotton mitogenomes. Known protein-coding genes, tRNA and rRNA genes, and gene fragments are shown on the line. Genes on the right side and left side of the line are transcribed direct and inverted, respectively. Colors indicate genes by function: Complex I (*nad*; yellow), Complex II (*sdh*; green), Complex III (*cob*; yellowish green), Complex IV (*cox*; light pink), Complex V (*atp*; olive-green), ribosomal proteins (brown), maturase (matR; orange), other genes (*ccm* and tRNA; purple), intron (white)

Cotton is the first species that the mitogenome is sequenced among the large numbers of malvales. We performed de novo sequencing of three mitogenomes lines: a) CMS-2074A, b) CMS-2074S, and c) E5903 (a restorer line). Lines 2074A and E5903 were derived from integrating the cytoplasm of diploid species *G. harknessii* (CMS-D_2–2_) into tetraploid *G. hirsutum*; while, 2074S was a result of alloplasmic *G. hirsutum* with *G. hirsutum* L. CMS-AD_1_-derived cytoplasm [39]. The mitogenomes of the three lines were highly conserved with the sequence identity more than 96%, indicating the preservation of the mitochondrial genome during cross-breeding (Table 1; Fig. 1). The mitogenomes of the three lines were 666,081 bp (E5903), 668,584 bp (2074A) and 668,464 bp (2074S), and there was about 3 kb difference detected (Table 1). These observations were close to the previous estimations based on restriction digestion patterns (690 kb – 710 kb) [56, 57]. Compared to the maintainer line 2074B, the above three lines (E5903, 2074A and 2074S) had more repeats. In four lines, the mitogenomic sequences belonging to the coding genes (including duplicated genes) and the plastid-derived sequences varied by less than 1% (Table 1, Additional file 4: Table S3). Both the proportions of nuclear-derived intergenic sequences and large repeats varied by 1–2%. Notably, the two CMS lines, 2074A and 2074S, contained two large inverse and direct repeats. Overall, the three mitogenomes, 2074A, 2074S, and E5903, had similar syntenic arrangements and were 87% identical in sequences’ similarity with the maintainer line, indicating general conservation among the varieties within species.

**Table 1:** Main features of the assembled *Gossypium* mitogenomes

Genome Characteristics	2074A	2074S	2074B	E5903
Genome size (bp)	668,464	668,584	621,884	666,081
GenBank ID	JX536494.1	JX944505.1	JX065074.1	JX944506.1
Circular chromosomes	1	1	1	1
Percentage G + C content (%)	44.97	44.98	44.98	44.95
Protein genes	37	37	36	37
tRNA genes	30	30	29	30
Native	18	18	17	18
Plastid-derived	12	12	12	12
tRNAs with introns	3	3	3	3
rRNA genes	4^a^	4^a^	4^a^	4^a^
Genic content percent coverage of total genome				
Exonic	6.23	6.23	6.25	6.39
Intronic-c	4.43	4.43	4.45	4.76
Intergenic content percent coverage				
Chloroplast-derived	1.43	1.44	1.35	1.37
Nuclear-derived	8.44	8.83	7.11	8.29
Repeat content percent coverage of total genome				
Large repeats: > 1 kb(number)	11.78 (4)	11.74 (5)	9.44 (4)	11.32 (7)
Small repeats: < 1 kb(number)	4.71 (475)	4.77 (476)	4.05 (465)	4.81 (470)

^a^Present *rrn26* has two copies

The main cycle (MC) molecules of two male sterile lines (2074A and 2074S) are 47 kb larger than that of the maintainer line (2074B). The reason is that they contain three large repeats, for examples, one is 10 kb larger than that of 2074B, which are similar as observed in the sterile line Ks3 that contains four repeats larger than 20 kb [41], and the sterile line TK18-MS that contains a pair of repeats of 86 kb in its MC molecule [58]. The intergenic regions of plant mtDNAs often contain retrotransposons from nuclear [25], chloroplast [59, 60] and other plant mitogenome [28]. 2074A and 2074S have more retrotransposons than 2074B and E5903, and they contain six unique regions with a total length of 31,694–36,741 bp. Furthermore, these sequences are novel and most are located in the intergenic regions, showing that they have a faster rate of evolution as similarly reported by Palmer et al., 2000 [61].

### Gene evolution among CMS, restorer, and maintainer lines

Cotton mitogenomes typically contain 36 genes (Table 2; Fig. 1) whose content is largely conserved among species and across angiosperms, with few differences (for example, contrary to other sequenced plants, *rpl2* in cotton lacks an intron [42, 43, 58]). As the case in the mitogenomes of other angiosperms, most of the genes encode proteins involved in ATP-generating electron transport: nine subunits of complex I (*nad* 1, *nad* 2, *nad* 3, *nad* 4, *nad* 4 L, *nad* 5, *nad* 6, *nad* 7 and *nad* 9), two subunits of complex II (*sdh* 3 and *sdh* 4), one subunit of complex III (*cob*), three subunits of complex IV (*cox* 1, *cox* 2 and *cox* 3), and five subunits of complex V (*atp* 1, *atp* 4, *atp* 6, *atp* 8 and *atp* 9); four genes involved in biogenesis of cytochrome c (*ccm* C, *ccm* B, *ccm* FC and *ccm* FN); 10 genes encode for ribosomal proteins (*rpl* 2, *rpl* 5, *rpl* 10, *rpl* 16, *rps* 3, *rps* 4, *rps* 7, *rps* 10, *rps* 12 and *rps* 14), while the numbers of the ribosomal proteins are usually variable among different species [58]. In addition, one gene (*mttB*) is involved in independent membrane targeting and translocation system, and one maturase gene (*matR*) is mapped within the 4th intron of *nad*1. 9 protein-encoding genes (*ccmFC*, *cox2*, *rps10*, *rps3*, *nad1*, *nad2*, *nad4*, *nad5* and *nad7*) contain a total of 23 group II introns, among of which three are trans-spliced (*nad* 1, *nad* 2 and *nad* 5). As previously noted, the cotton mitogenomes presented here lack *rps1*, *rps2*, *rps11*, *rps13*, *rps19* and *sdh2*, and only partial sequences of these genes were detected in cotton mitogenomes.

**Table 2:** Gene contents of *Gossypium* mitotypes

Product group	Gene	2074A	2074S	2074B	E5903	Product group	Gene	2074A	2074S	2074B	E5903
complex I	*nad1*	+	+	+	+	Ribosome	*rps3*	+ 2^b^	+ 2	+ 2	+/ψ-
	*nad2*	+	+	+	+		*rps4*	+	+	+	+
	*nad3*	+	+	+	+		*rps7*	+	+	+	+
	*nad4*	+	+	+	+		*rps10*	+	+	+	+
	*nad4L*	+	+	+	+		*rps12*	+	+	+	+
	*nad5*	+	+	+	+		*rps14*	+	+	+	+
	*nad6*	+	+	+	+		*rpl2*	+	+	+	+
	*nad7*	+	+	+	+		*rpl5*	+	+	+	+
	*nad9*	+	+	+	+		*rpl10*	+	+	+	+
complex II	*sdh3*	+	+	+	+		*rpl16*	+	+	+	+
	*sdh4*	+	+	+	+	tRNA	*trnC(GCA)-cp*	+	+	+	+
complex III	*Cob*	+	+	+	+		*trnD(GUC)-cp*	+ 2	+ 2	+ 2	+ 2
complex IV	*cox1*	+	+	+	+		*trnE(UUC)*	+	+	+	+
	*cox2*	+	+	+	+		*trnF(GAA)*	+ 2	+ 2	+ 2	+ 2
	*cox3*	+	+	+	+		*trnfM(CAU)-cp*	+ 4	+ 4	+ 4	+ 4
complex V	*atp1*	+	+	+	+		*trnG(GCC)*	+	+	+	+
	*atp4*	+	+	+	+		*trnH(GUG)-cp*	+	+	+	+
	*atp6*	+	+	+	+		*trnK(UUU)*	+	+	+	+
	*atp8*	+	+	+	+		*trnM(CAU)*	+ 2	+ 2	+ 1	+ 2
	*atp9*	+	+	+	+		*trnI(UAU)*	+	+	+	+
Cytochrome C	*ccmB*	+	+	+	+		*trnN(GUU)-cp*	+	+	+	+
	*ccmC*	+	+	+	+		*trnP(UGG)*	+ 3	+ 3	+ 3	+ 3
	*ccmFN*	+	+	+	+		*trnQ(UUG)*	+	+	+	+
	*ccmFC*	+ 2^a^	+ 2	+	+ 2		*trnS(GCU)*	+ 2	+ 2	+ 2	+ 2
Other gene	*mttB*	+	+	+	+		*trnS(GGA)-cp*	+	+	+	+
	*matR*	+	+	+	+		*trnS(UGA)*	+	+	+	+
rRNA	*rrn5*	+	+	+	+		*trnSup(UUA)*	+	+	+	+
	*rrn18*	+	+	+	+		*trnV(GAC)*	+	+	+	+
	*rrn26*	+ 2	+ 2	+ 2	+ 2		*trnW(CCA)-cp*	+ 2	+ 2	+ 2	+ 2
							*trnY(GUA)*	+	+	+	+

Note. −+, denotes present; −, denotes absent; ^a^Gene copy number is shown after +; ^b^*rps3–2*is a pseudo gene

As reported previously, several mitochondrial genes exist in repeat regions and in multiple copies (Additional file 5: Table S4). However, unlike the mitogenome of *G. hirsutum* maintainer line, those of 2074A, 2074S and E5903 contain duplicated *trnM* (CAU) and *ccmFC* in large repeats. In addition, congruent with prior results, *rps3* is located at a repeat’s boundary and varied in structure among these four *Gossypium* mitogenomes. In cotton, *rps3* contains a central domain (pfam00013) which has been lost in the incomplete duplicates (pseudogene *rps3–2*) of 2074A, 2074S, and 2074B.

A total of 47 SNPs exists within 21 protein-coding genes in four mitogenomes analyzed, and only 11 SNPs were synonymous mutations (1 in 2074A mitogenome and 10 in 2074B mitogenome, Table 3). Remarkably, the numbers of nonsynonymous mutations (36 SNPs) are over three times as that of synonymous mutations, and nonsynonymous mutations were nearly evenly distributed among the mitochondrial genomes (10, 10, 9, and 7 unique nonsynonymous SNPs in 2074A, E5903, 2074S and 2074B, respectively). Most of these SNPs represent transversions rather than transitions (29 versus 7), and many of them were found in ribosomal protein-coding genes, (i.e., *rpl2*, *rpl5*, *rpl10*, *rpl16*, *rps3*, *rps4* and *rps10*; Table 3). As protein-coding genes are extraordinarily conserved and exhibit slow evolutionary rates, the abundance of non-synonymous changes reported here may represent CMS-related candidate genes, although this needs to be functionally verified in each case. Mitochondrial genes, *rps3* and *rpl2,* separately containing 3 and 2 nonsynonymous changes between the mitogenomes of 2074A (and 2074S)/E5903 and 2074B, might represent suitable candidates.

**Table 3:** The protein variation in four *Gossypium* mitogenomes

Gene	Len	Var	IDY	Loc	2074A		2074S		E5903		2074B		NSM	SM	aa-Var
					N-S	P-S	N-S	P-S	N-S	P-S	N-S	P-S			
*atp4*	585	1	99.8	222	tt**T**	Phe	tt**T**	Phe	tt**T**	Phe	tt**C**	Phe	1	0	
*atp8*	465	1	99.8	171	ag**A**	***Arg***	ag**A**	***Arg***	ag**A**	***Arg***	ag**C**	Ser	0	3	Ser-Arg
*atp9*	225	1	99.6	27	gg**A**	Gly	gg**T**	Gly	gg**T**	Gly	gg**T**	Gly	1	0	
*ccmB*	621	1	99.8	11	c**A**t	***His***	c**T**t	Leu	c**T**t	Leu	c**T**t	Leu	0	1	Leu-His
*ccmFC*	1323	1	99.8	585	gt**C**	Val	gt**C**	Val	gt**C**	Val	gt**G**	Val			
*cox1*	1593	3	99.8	415	**A**cc	Thr	**A**cc	Thr	Ccc	***Pro***	**A**cc	Thr			Thr-Pro
				960	at**A**	Ile	at**A**	Ile	at**A**	Ile	at**C**	Ile			
				1428	at**A**	Ile	at**A**	Ile	at**A**	Ile	at**C**	Ile	2	1	
*cox2*	783	1	99.9	481	**T**ta	Leu	**T**ta	Leu	**T**ta	Leu	**C**ta	Leu	1	0	
*cox3*	798	4	99.6	157	**C**tc	Leu	Atc	**Ile**	Atc	***Ile***	**C**tc	Leu			Leu-Ile
				294	tt**T**	Pro	tt**T**	Pro	tt**T**	Pro	ttG	***Leu***			Pro-Leu
				295	**G**ct	Ala	**G**ct	Ala	**G**ct	Ala	Tct	***Ser***	0	4	Ala-Ser
*matR*	1968	1	99.9	1858	**A**aa	***Lys***	**A**aa	***Lys***	**A**aa	***Lys***	Caa	Gln	0	3	Gln-Lys
*nad2*	1467	1	99.9	783	tc**G**	Ser	tc**G**	Ser	tc**G**	Ser	tc**T**	Ser	1	0	
*nad3*	357	1	99.7	317	t**C**t	Ser	t**C**t	Ser	t**C**t	Ser	tTt	***Phe***	0	1	Ser-Phe
*nad4*	1488	3	99.8	33	ga**T**	Asp	ga**T**	Asp	ga**T**	Asp	ga**C**	Asp			
				240	at**C**	Ile	at**C**	Ile	at**C**	Ile	at**A**	Ile			
				242	a**A**t	Asn	a**A**t	Asn	a**A**t	Asn	aTT	***Ile***	2	1	Asn-Ile
*nad7*	1185	1	99.9	24	at**C**	Ile	at**C**	Ile	at**C**	Ile	at**A**	Ile	1	0	
*rpl2*	1005	2	99.8	45	tt**G**	***Leu***	tt**G**	***Leu***	tt**G**	***Leu***	ttT	Phe			Phe-Leu
				292	**C**tc	***Leu***	**C**tc	***Leu***	**C**tc	***Leu***	Atc	Ile	0	6	Ile-Leu
*rpl5*	582	1	99.8	139	**C**aa	***Gln***	**C**aa	***Gln***	**C**aa	***Gln***	Aaa	Lys	0	3	Lys-Gln
*rpl10*	489	1	99.8	361	**A**aa	***Lys***	**A**aa	***Lys***	**A**aa	***Lys***	Gaa	Glu	0	3	Glu-Lys
*rpl16*	435	1	99.5	270	gt**C**	Val	gt**C**	Val	gt**C**	Val	gt**A**	Val	1	0	
*rps3*	1707	3	99.8	1670	a**A**g	Lys	a**A**g	Lys	a**A**g	Lys	aGg	***Arg***			Lys-Arg
				1676	g**GA**	Gly	g**GA**	Gly	g**GA**	Gly	gAC	***Asp***			Gly-Asp
				1678	**C**gt	Arg	**C**gt	Arg	**C**gt	Arg	Ggt	***Gly***	0	3	Arg-Gly
*rps4*	1098	1	99.9	535	**C**aa	***Gln***	**C**aa	***Gln***	**C**aa	***Gln***	Aaa	Lys	0	3	
*rps10*	333	1	99.4	311	gTC	***Val***	g**AA**	Glu	g**AA**	Glu	g**AA**	Glu	0	1	Glu-Val
*sdh3*	435	1	99.8	33	tt**C**	***Phe***	tt**C**	***Phe***	tt**C**	***Phe***	ttA	Leu	0	3	
nonsynonymous mutation		31			10		9		10		7	total	10	36	17
Synonymous mutation					1		0		0		10				

Note. –Len, length of gene CDS sequence; *Var* variant sites in fourmitogenomes, *IDY* identity of gene CDS sequences, *Loc* location of variant sites, *N-S* nucleotide sequence, *P-S* amino acid sequence, *Boldface*, variant nucleotide, *Bold italic* variant amino acids, *NSM* nonsynonymous mutation, *SM* synonymous mutation, *aa-Var* amino acidvariation, Boldface mark is mutated base and amino acid

In the four mitogenomes analyzed, we found many gene editing events, for example, ACG was edited into AUG as start codon in three genes (*rps10*, *nad1* and *nad4L)*, and AUU was modified into AUG in one gene (*mttB*). There were five cases where gene editing generated stop codon, thereinto, four cases were the conversion of TAG into CGA in *rps10*, *ccmC*, *atp9* and *ccmFC* genes; however, TAG was converted to CAA in *atp6* gene. of Evolutionary rates analysis (ka/ks or ω) revealed that the ratios of ka to ks of nine genes (*rps12*, *matR*, *atp1*, *mttB*, *rps4*, *rrn18* and *nad1*) were greater than 1, which implied a positive selection. In addition, that of two genes (*rpl5* and *cox3*) were less than 1, which implied a purifying selection. By contrast, the non-coding regions appeared to be rapidly diverged (Additional file 6: Table S5).

### Repeated sequences and unique sequences

**Fig. 2:**
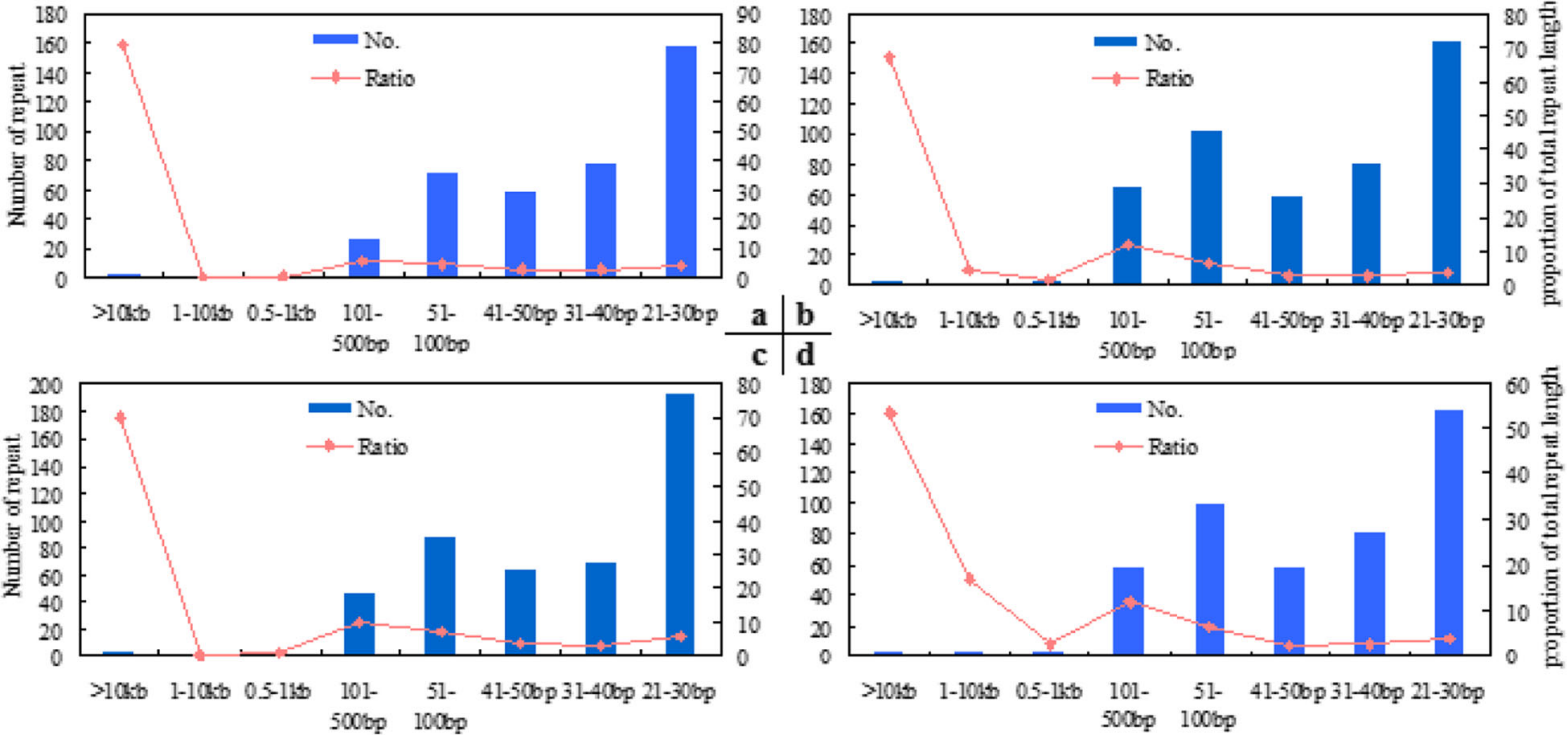
The size distribution of repetitive content by the number of repeat pairs and total repeat length. The X position is repeat size category, which contains more than 10 kb, 1–10 kb, 0.5–1 kb, 101–500 bp, 41–100 bp, 31–40 bp, 21–30 bp. The Y positions are number of repeats pairs (primary axis) and proportion of total repeat length (secondary axis). The (**a**), (**b**), (**c**), (**d**) present 2074A, 2074S, 2074B, E5903 mitogenome, respectively

The plant mitogenomes harbor massive repeated sequences, and the genome sizes tend to increase the genomic coverage by large repeats [62–65]. Our analysis revealed duplications were the main reason for the difference in size among the four lines. The duplicate lengths varied from 504 bp to 29 kb, which constituted 9.4–12.0% of the total genome lengths (Table 4). Two duplicated fragments 11,191 bp and 10,632 bp were present in the mitogenomes of all four lines. There was a common duplicate in three mitogenomes of 2074A, 2074S and E5903, but it was absent in 2074B. The mitogenomes of 2074A and 2074S were mostly identical, with one exception that a repeat sequence was present in 2074A but absent in 2074S. The mitogenome of 2074A is made up of a repeat sequence (29,277 bp), whereas that of 2074S consists of two repeat sequences (24,378 and 4621 bp) that are separated by a gap (Table 4). Total backbone DNA sequences represented a concatenation containing all basic fragments among all mitogenomes. When considering only one copy of each duplicated sequence, we found the genomic variations became small, especially from the same origin. The sizes of the backbone mtDNA sequences of 2074A and E5903 are same, and have a minimal difference with that of 2074S. Other repeats are smaller in size, and distribute distinctly and vary in copy number (Fig. 2).

**Table 4:** Length and percentage of duplicated fragments (up to 500 bp)

Genome	Genome length (bp)	Duplication length (bp)^a^	% of in genome	Minimal length (bp)^b^	Maximal. length (bp)^b^	Number of fragments	Genome length without duplication (bp) (Percentage)
2074A	668,464	80,545	12.0	504	29,277	7	587,919 (87.95%)^c^
2074S	668,584	80,269	12.0	505	27,666	8	588,315 (87.99%)
2074B	621,884	58,734	9.4	879	27,558	5	563,150 (90.56%)
E5903	666,082	78,161	11.7	504	21,563	11	587,921 (88.27%)

Note. –^a^All duplicated copies less one; ^b^Length of one copy; ^c^% of backbone fragments in genome

Intra-genomic recombination is an active phenomenon in the mitogenomes of plants [25]. Recombination frequency depends on the size of repeats, for example, large and direct repeats (> 1 kb) are associated with homologous recombination that will lead to the formation of sub-genomic molecules [66]. These four cotton mitogenomes exist as 4–7 larger repeats that produced subcircles. In 2074A, two pairs of subcircles are mediated by direct-repeat AR1, whereas one MC genomic circle may be produced by inverted-repeat AR4. However, 8–12 positive Fosmid clones covered all these large repeats and nine positive Fosmid clones covered all these repeats in E5903, which suggests that these four big repeats didn’t formed subcircles in 2074A and E5903 mitogenomes (Additional file 7: Figure S1). Importantly, every nodal point of large repeats is verified by long-PCR with special primers designed from their sequences (the PCR products were overlapped with repeats and non-repeats regions more than 300 bp, Additional file 2: Table S2A, Additional file 3: Table S2B).

Cytoplasmic male sterility is frequently associated with novel, chimeric, and often disruptive ORFs [3, 64]. In this study, we evaluated the mitogenomes of two CMS lines in the context of their maintainer and restorer lines for unique sequences that contain novel coding regions responsible for sterility in *G. hirsutum*. Six unique sequences (U1 to U6) were similar in two CMS lines, but were absent in the maintainer and restorer lines (Table 5). The total lengths of these regions were 31,694 bp in 2074A and 36,741 bp in 2074S, respectively (Table 5). Overall, these regions were unique with little similarities to known mitochondrial and nuclear sequences of other plants. These unique regions were frequently associated with repeats’ boundaries, which might indicate an origin from new sequence migration and recombination.

**Table 5:** The unique sequences in 2074A and 2074S compared with 2074B

No.	Location^a^	Length(bp)	Joint of syntenic regions	Predicted ORF^b^	Identity sequences^d^
U1	1–5156	5156	S1	Aorf1, Aorf2, Aorf3; Sorf1, Sorf2, Sorf3	2316–2885, 3107–3879 *Carica papaya* mitochondrion.
U2	16,918-17,305	388	S2-S3		236–379 *Citrullus lanatus* mitochondrion, *Beta vulgaris subsp.* 5 aponica genotype male-sterile E mitochondrion.
U3	143,667–151,556 (143,674-151,564)	7890	S6, S5-S7	Aorf7, Sorf7	2888–3734, 7191–7409, 6160–6348 *Vigna 5 aponic* mitochondrion, *Malus x domestica* mitochondrion, *Beta vulgaris* subsp. 5 aponica genotype male-sterile G mitochondrion.
U4	237,227- 238,728 (237,182–238,681)	1502	S10		893–1502, 413–720, *Phoenix dactylifera* mitochondrion, *Nicotiana tabacum* mitochondrion.
U5^c^	438,450–457,430 (443,399-457,334)	18,981 (13936)	S14, S13-S15	Aorf18, Aorf19, Aorf20, Aorf21, Aorf22; Sorf17, Sorf16, Sorf18, Sorf19, Sorf20, Sorf21	6486–7727, *G. hirsutum* clone MX008C17-jmb, 5270–5431, 5610–5890, 10,317–11,602, 12,127–12,482 *Mimulusguttatus* mitochondrion; 2286–2703 unknown chloroplastid sequence. 15,875–18,884 *O. berteriana* mtDNA for *rps3* and *rpl16*.
U6	665,761 -668,584 (665,642-668,464)	2824	S22	Aorf29; Sorf30	565–857, 1918–2188, 879–1159, *Phoenix dactylifera* mitochondrion, *Lotus 5 aponicas* strain MG-20 mitochondrion.

Note. –^a^ figures in brackets denote the sites in 2074A mitogenome; ^b^there are 5 ORFs predicted in U5; ^c^U5 is 13936 bp in 2074A, and is longer 5148 bp at 3’end sequence in 2074S ^d^the identity is more than 80%, the figures denote the sites of alignment fragments

### Mitochondrial genome syntenic evolution and organization

In addition, compared with 2074A, we analyzed the syntenic evolution of 4 cotton mitogenomes. We found 22 syntenic regions (named as S1 - S22), ranging from 2824 to 147,683 bp, which possessed at least 98% identity (Additional file 8: Figure S2). 2074B has lost syntenic segments S1 (U1), S6 (U3), S14 (U5), and S22 (U6). However, some segments are conserved in four mitogenomes, such as S5 - S6, S8 - S9, S10 - S13 and S15 - S20; the terminal sequences of S3, S4, S7, S13 and S21 are four large repeats (AR4, AR3, AR1, AR2, AR1, respectively), and the former sequence S20 was AR1 (as the difference of these larger repeats). The syntenic regions are broken, which suggests the repeat sequences are more dynamic and have undergone recombination in breeding process. S10 and S2 are broken by unique sequences U4 and U2, while other syntenic regions are more or less interrupted by insertion or deletion. These two cytoplasmic male sterile lines are more complex in nucleotide sequence composition, which suggests that male sterility may have been favored by faster rates of rearrangement and evolution, or CMS itself might have caused faster rearrangement and evolution.

### CMS in two cytoplasmic male sterile lines of *Gossypium hirsutum*

CMS is a widespread phenomenon in plants and is associated with abnormal mitochondrial ORFs [7]. The occurrence of male sterility is an important feature in cotton breeding system. CMS is expected to be affected by mitochondrial gene(s), ORF content(s) and diversity during the emergence and selection of CMS specific mitochondrial genes. In other plants, several CMS-associated aberrant genes are located upstream or downstream of certain known genes and co-transcribed together [7, 67]. Since novel ORFs may be relevant to CMS, we analyzed all the predicted ORFs about their origin, conservation, function and expression. We compared all ORFs of 2074A and 2074S with that of the maintainer line 2074B, we observed 28 and 30 novel ORFs in 2074A and 2074S, respectively (Tables 6 and 7). The ORFs of 2074A were named as *Aorf1* to *Aorf28*, and *Aorf4* was duplicated in 2074A; while, those of 2074S were named as *Sorf1* to *Sorf30*, and *Sorf4* also was duplicated in 2074S. 11 of the ORFs are common in 2074A and 2074S. The length of polymorphisms in ORFs was frequently caused by frame shift mutations with several nucleotides’ insertions/deletions.

**Table 6:** Chimeric ORFs (> 300 bp) presented in 2074A mitogenomes

2074 AORF	Start	End	Strand	Length (bp)	2074B	E5903	Tra-dom^c^	Uni/R-seq^d^	Homologous sequence^e^	RNA-Seq Log2	
										2074B/2074A	F1-A/2074A
Aorf1	3872	4483	+	612	-^a^	*^b^	1	U1	No homologous sequence	−11.76	2.73
*Aorf2*	4461	3700	–	762	–	*	6	U1	583–759, 86%, papaya mtDNA	−12.58	2.62
Aorf3	5180	4878	–	303	–	*	0	U1	154–301, 96%, papaya mtDNA	− 14.54	2.52
*Aorf4*	27,402	26,845	–	558	Some	some	1	AR4(524)	45 bp, *rps3*(1–45)^f^; 47 bp *sdh3*	−0.22	2.92
*atp8*	28,431	27,967	–	463	*	*				−0.35	2.56
Aorf5	86,768	87,067	+	300	–	–	0		98%, other plant mtDNA	−11.92	3.33
Aorf6	143,074	142,757	–	318	Some	–	0		1–301, 95%, *Phoenix ductylifera* mtDNA	0.00	12.63
Aorf7	144,192	144,596	+	405	–	*	0	U3	No homologous sequence	0.00	9.96
Aorf8	149,251	148,952	–	300	–	*	0	U3	4–242, 94%, *Beta vulgaris* mtDNA	−13.47	2.25
*rrn26*	185,940	189,313	+	3374	*	*				1.26	3.17
*Aorf9*	189,332	189,688	+	357	Some	some	1	AR1(7125)	76 bp, *nad7*	−0.70	2.41
Aorf10	258,310	257,840	–	471	Some	some	1	up AR2 93 bp	4–424, 92%, *Ricinus communis* mtDNA	− 0.25	2.66
Aorf11	314,879	315,265	+	387	Some	some	0		43 bp, *nad7*	−1.00	2.63
Aorf12	323,440	323,838	+	399	Some	some	0		No homologous sequence	−12.06	2.01
*rps14*	324,431	324,751	+	321	*	*				−1.22	2.09
*cob*	326,094	327,256	+	1163	*	*				−0.91	3.37
Aorf13	335,286	334,948	–	339	Some	some	0		No homologous sequence	1.26	3.17
Aorf14	343,549	343,223	–	327	–	–	0		36 bp, *cox2ex1*	−1.25	3.38
*rpl2*	346,637	347,641	+	1005	*	*				−0.27	2.05
*Aorf4–2*	348,085	348,642	+	558	Some	some	1	AR4(524)	45 bp, *rps3*; 47 bp, *sdh3*	−0.22	2.92
Aorf15^h^	388,830	388,447	–	384	Some	some	1		No homologous sequence	−1.08	2.79
Aorf16	397,394	397,735	+	342	–	–	0		No homologous sequence	−1.64	3.41
Aorf17	415,813	415,265	–	549	Some	some	0	up AR2 23 bp	39–527, 94%, *Citrullus lanatus* mtDNA	−11.92	3.33
Aorf18	449,399	449,001	–	399	–	some	0	U5	123–393, 82%, tobacco mtDNA	−11.06	4.57
Aorf19	452,355	453,074	+	720	–	*	2	U5	No homologous sequence	0.00	15.49
Aorf20	452,473	452,781	+	309	–	*	1	U5	No homologous sequence	0.00	13.35
Aorf21	454,116	453,781	–	336	–	*	0	U5	93%, *Mimulus guttatus* mtDNA	0.00	14.48
Aorf22	454,900	454,451	–	450	–	*	0	U5	96%, *Mimulus guttatus* mtDNA	−10.88	3.10
Aorf23	465,398	465,751	+	354	Some	some	0		167–331, other plant mtDNA	0.09	2.15
Aorf24	490,816	491,292	+	477	–	–	2		64 bp, *rps4*	−2.19	3.93
Aorf25^g^	491,321	491,689	+	369	Some	*	0		20 bp, *ccmFC*	−3.15	4.83
Aorf26	508,562	507,753	–	810	Some	some	0		306–479, other plant mtDNA	1.45	5.21
*cox1*	631,928	633,518	+	1591	*	*				−0.32	1.86
*Aorf28*	633,740	634,606	+	867	Some	–	0	up AR1 1760 bp	56 bp, *atp4*(1–56)^f^	−1.47	3.02
*cox3*	634,937	635,734	+	798	*	*				−0.03	2.69
Aorf27	665,520	666,155	+	636	–	*	0	AR1, U6	No homologous sequence	−4.71	0.35

Note. –^a^no detected; *^b^have this ORF; ^c^Tra-dom: transmembrane domain; ^d^Uni/R-seq: unique sequence or repeat sequence; ^e^Homologous sequence contains the sequence of genes in cotton and mitochondrial sequences of other plants; ^f^ Aorf4 contain a fragment that is 1-45 bp of *rps3*, Aorf28 contain a fragment that was 1-56 bp of *atp4,* identity is 100%; ^g^Aorf25 is in upstream 70 bp of *nad5ex4*; ^h^the end of Aorf15 is longer 81 bp than Sorf14

**Table 7:** Chimeric ORFs (> 300 bp) presented in 2074S mitogenomes

2074S	Length (bp)	Tra-dom^c^	Uni/Rep-seq^d^	2074B	E5903	Location	Homologous sequence^e^
Sorf25	660	-^a^		–	*^b^	down *atp4* 2192 bp	19 bp, *ccmFN*
Sorf16	381	1	U5	–	–		157 bp, *rps3ex2*
Sorf1	612	1	U1	–	*		No homologous sequence
Sorf26	810	–		partial	Partial		306–479, other plant mtDNA
Sorf21	450	–	U5	–	*		96%, *Mimulus guttatus* mtDNA
Sorf20	336	–	U5	–	*		93%, *Mimulus guttatus* mtDNA
Sorf17	399	–	U5	–	partial ^e^		123–393, 82%, tobacco mtDNA
Sorf15	549	–	up SR2	partial	Partial		39–527, 94%, *Citrullus lanatus* mtDNA
Sorf7	405	–	U3	–	*		No homologous sequence
*Sorf14*	315	1		partial	Partial		No homologous sequence
Sorf13	327	–		–	–		36 bp, *cox2ex1*
Sorf12	339	–		partial	Partial		No homologous sequence
Sorf9	471	1	up SR2	partial	Partial		4–424, 92%, *Ricinus communis* mtDNA
Sorf6	318	–		partial	–		1–301, 95%, *Phoenix ductylifera* mtDNA
*Sorf8*	357	1	SR1	partial	Partial	down *rrn26* 19 bp	76 bp, *nad7*
*Sorf4*	558	1	SR4	partial	Partial	down *atp8* 565 bp	45 bp, *rps3*(1–45)^f^; 47 bp *sdh3*
*Sorf4–2*	558	1	SR4	partial	Partial	up *rpl2* 444 bp	45 bp, *rps3*; 47 bp, *sdh3*
Sorf3	303	–	U1	–	*		154–301, 96%, papaya mtDNA
Sorf2	762	6	U1	–	*		583–759, 86%, papaya mtDNA
Sorf10	387	–		partial	Partial		43 bp, *nad7*
Sorf11	399	–		partial	Partial		No homologous sequence
Sorf19	309	1	U5	–	*		No homologous sequence
Sorf18	720	2	U5	–	*		No homologous sequence
Sorf5	300	–		–	–		98%, other plant mtDNA
Sorf22	354	–		partial	Partial		167–331, other plant mtDNA
Sorf23	477	2		–	–		64 bp, *rps4*
*Sorf24*	369	–		partial	*	up *nad5ex4* 91bp^g^	20 bp,*ccmFC*
*Sorf29*	867	–	up SR1	partial	–	down *cox1* 222 bp, up *cox3* 331 bp	56 bp, *atp4*(1–56)^f^
Sorf30	636	–	SR1, U6	–	*		No homologous sequence
Sorf28	414	–		partial	Partial		No homologous sequence
*Sorf27*	951	3		partial	Partial	down *rrn5* 46 bp	No homologous sequence

Note.–^a^no detected; *^b^have this ORF; ^c^Tra-dom: transmembrane domain; ^d^Uni/R-seq: unique sequence or repeat sequence; ^e^Homologous sequence contains the sequence of genes in cotton and mitochondrial sequences of other plants. ^f^The similarity between 1–45bp in Sorf4 and 1–45bp in rps3, 1–56bp in Sorf29 and 1–56bp is 100%; ^g^ nad5ex4 is located at 91bp upstream of Sorf24

We categorized the specific ORFs into three basic groups: I) ORFs near the functional genes, which is transcribed in the same direction with adjacent positioned genes either up or down stream, and could be co-transcript relevant to CMS (*Aorf4, Aorf25, Aorf27, Aorf28, Sorf4, Sorf8, Sorf14, Sorf27* and *Sorf28*); 2) Special ORFs in unique regions of sterile lines, which always have short-sequences homology to chloroplast or mitochondrial sequences of other plants; such as *Aorf2, Aorf18, Sorf15, Sorf16* and *Sorf2* that were found in unique sequences of two sterile lines. Mostly, they are similar to chloroplast or mitochondrial sequences of other plants, or have no homology sequences in NCBI-NR database. In the third group, the ORFs are comprised of homologous sequences of 2074B and unique sequences such as *Aorf14, Sorf13* and *Sorf14*.

**Fig. 3:**
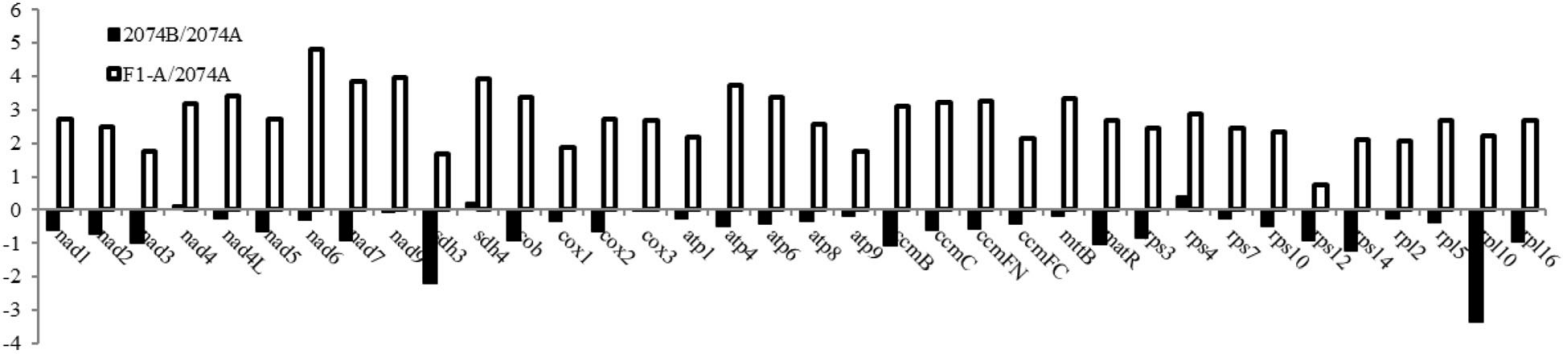
Differential expression of mt genes in 2074A, 2074B and F_1_-A. Log2 transformations of the expression fold changes (2074B/2074A and F_1_-A/2074A) are represented by bars. Y axis denotes the levels of transformed expression fold changes

To further verify whether these ORFs were functionally associated with CMS, we profiled the expression of mitochondrial genes and ORF’s based on RNA-seq data of flower buds (3–5 mm in size) in CMS 2074A, maintainer 2074B, and the fertility-restored F_1_ (2074A × E5903). Among all the three lines, the expression of mitochondrial genes was highest in F_1_ and lowest in 2074B (Fig. 3, *P* < 0.05). The expression levels of *shd3* and *rpl10* genes were higher in 207A than in 2074B (Fig. 3, *P* < 0.05). Taking the sequences of 28 predicted ORFs in 2074A as a pool; we used Blastn to match all three-transcript data (2074A, 2074B and F_1_). As a result, 10 ORFs were expressed at high levels (10 fold) as compared to the similar sequence (with 1–3 gap) in 2074B; five ORFs were expressed at high levels as compared to the similar sequence in F_1_; the five ORFs were not expressed in 2074B (Additional file 9: Figure S3). Based on the first group principle, the ORFs near to functional genes, we found that four pairs of ORFs and their nearby genes (*Aorf4* and *atp8*, *Aorf9* and *rrn26*, *Aorf4–2* and *rpl2*, *Aorf28* and *cox1*/*cox3*) have same expression trend both in 2074B/2074A and F_1/_2074A, therefore, these four ORFs might be co-transcribed with functional genes and relevant to CMS.

**Fig. 4:**
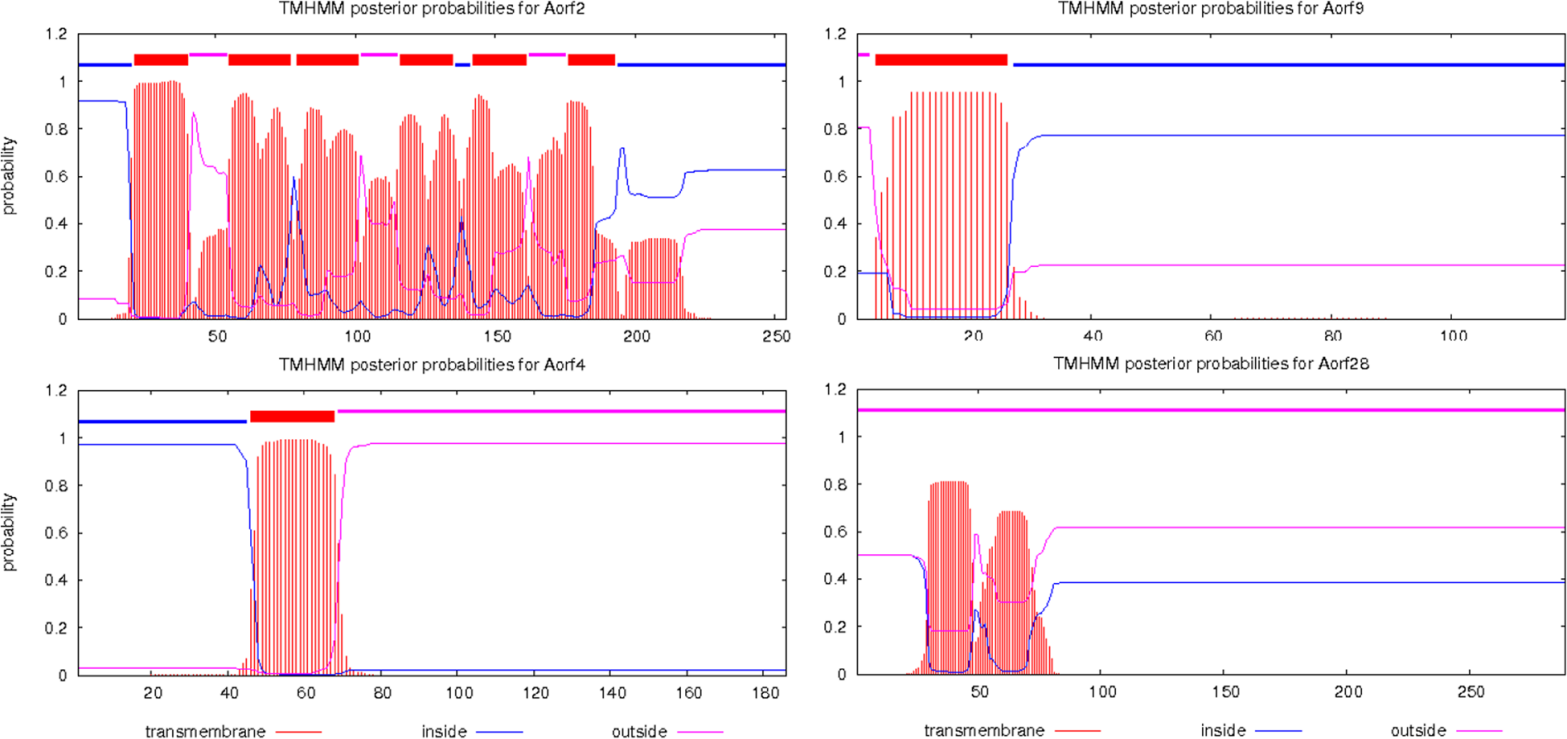
The probability of transmembrane domains of *Aorf4, Aorf9, Aorf2* and *Aorf28* gene products

Furthermore, we analyzed 16 reported CMS-associated ORFs. We found that these ORFs (78–488 bp) are near to co-transcribed genes and form a bicistronic complex with many functional genes. In this context, six ORFs in CMS2074S (*Sorf25, Sorf4, Sorf4–2, Sorf29, Sorf8* and *Sorf27*) and five ORFs in CMS2074A (*Aorf12, Aorf4, Aorf4–2, Aorf28* and *Aorf9*) were close to functional genes within 565 bp, and six (*Sorf4, Sorf29, Sorf8, Aorf4, Aorf28* and *Aorf9*) of them are the products of rearrangements by large repeats. Additionally, these ORFs have transmembrane domain (except *Aorf28*, Table 5, Fig. 4) and same expression trend with their nearby genes. More important, four ORFs (*Aorf4, Aorf28, Aorf9* and *Aorf4–2*) and their functional genes (*atp8*, *cox1*, *cox3*, *rrn26* and *rpl2*) might have higher expression in CMS-2074A compared to F_1_. *Aorf4* (561 bp) is found at the downstream 565 bp of *atp8*. Besides, the first 45 bp of *Aorf4* are derived from *rps3*, while other partial sequences are identical to *sdh3* (the 5′-end of *orfH79* has 84 bp homology to *cox1*) and have same expression trends with *atp8* in 2074B/2074A (− 0.3) and F_1_-A/2074A (2.6~ 2.9). *Aorf4*–2 (561 bp) is found in the downstream 444 bp of *rpl2* and have same expression trends with *rpl2* in 2074B/2074A (− 0.2~ 0.5) and F_1_-A/2074A (2.9~ 3.2). *Aorf28* (867 bp), located at the downstream 241 bp of *cox1* and the upstream 311 bp of *cox3* (331 bp in 2074S), shows 66% identity with *Arabidopsis* mitogenome and is close to AR1. In addition, the expression trend of *Aorf28*, *cox1* and *cox3* were same. *Aorf9* (357 bp), located at the downstream 19 bp of *rrn26*, keeps same expression trends with *rrn26* in 2074B/2074A (− 0.7) and F_1_-A/2074A (2.4); as well, *Aorf9* also has 76 bp identity with *nad7* and 89% identity with *Ricinus* mitogenome. These four ORFs show the characters of CMS-associated genes and are similar to other ORFs, such as T-*urf13* of maize [14], S-*orf355*/*orf77* [66], *orf224* of rape [8, 68–70], *orf256* of wheat [15, 71], *orf125* of radish [72], etc. All above chimeric ORFs from other plants are always near and co-transcribed with functional genes, which makes functional genes transcribe improperly and causes abortion [73–76]. As to now, these results were only based on the genome and RNA-seq data, more experiments, including functional validation of overexpression or CRISPR/Cas9 these *orfs*, are needed to confirm the real CMS gene of upland cotton.

## Conclusions

The two almost identical male sterile lines, 2074A and 2074S, share high identity with the restore line E5903 but are different from their maintainer line 2074B, especially in non-coding regions. The cotton mtDNAs are 621,884–668,584 bp in length, and harbor 36 known protein-coding genes, three rRNAs (18S, 26S, and 5S rRNAs) as well as 18 different tRNAs. The rates of the coding genes (including duplicated genes) accounting for the total genomes’ length are almost similar, but the repeat sequences show a few differences. In addition, five genes (*rps1*, *rps2*, *rps13*, *rps19* and *sdh2*) have been lost and 38 nonsynonymous mutations occurred in 21 protein-coding genes, though they are functionally irrelevant. Out of 28 ORFs in CMS 2074A, four ORFs (*Aorf4, Aorf9, Aorf4–2* and *Aorf28*) are close to the functional genes and show similar characters to CMS-associated genes in other plants. These four ORFs may be the potential candidates conferring CMS in cotton.

## Additional files

Additional file 1: **Table S1.** Summary of the four mitogenomes sequencing and assembly. (DOCX 14 kb)

Additional file 2: **Table S2A.** The verification about breaking point of scaffolds between 2074A and 2074B. (DOCX 15 kb)

Additional file 3: **Table S2B.** The verification about breaking point of large repeats between 2074A and 2074B. (DOCX 15 kb)

Additional file 4: **Table S3.** The chloroplast-derived sequences (> 70 bp) found in four mitogenomes. (DOCX 19 kb)

Additional file 5: **Table S4.** List of multi-copy genes in cotton mtDNA. (DOCX 16 kb)

Additional file 6: **Table S5.** Nucleotide differences relative to the 2074B mitogenome. (DOCX 14 kb)

Additional file 7: **Figure S1.** The end sequencing positive clones in E5903 and 2074A. (DOCX 63 kb)

Additional file 8: **Figure S2.** The syntenic regions in four mitochondrial genomes. (DOCX 80 kb)

Additional file 9: **Figure S3.** Differential expression of CMS candidate ORFs in 2074A, 2074B and F1-A. Log2 transformations of the expression fold changes (2074B/2074A and F1-A/2074A) are represented by bars. Y axis denotes the levels of transformed expression fold changes. (DOCX 19 kb)

## Abbreviations

CMS: Cytoplasmic male sterility
cp: Chloroplast
G.: *Gossypium*
MC: Main cycle
mitogenome: Mitochondrial genome
mtDNA: Mitochondrial DNA
NGS: Next-generation sequencing technology
ORFs: Open reading frames
rRNAs: Ribosomal RNAs
tRNAs: Transfer RNAs
