# Supplementary material for: The comparison of four mitochondrial genomes reveals cytoplasmic male sterility candidate genes in cotton
Source: BMC Genomics. 2018 Oct 26;19:775. doi: 10.1186/s12864-018-5122-y (PMC6204043; doi:10.1186/s12864-018-5122-y)
Supplement: Supplementary file 1 — Table S1. Summary of the four mitogenomes sequencing and assembly. (DOCX 14 kb) [file 12864_2018_5122_MOESM1_ESM.docx]

**Supplemental information**

**Additional file 1:**


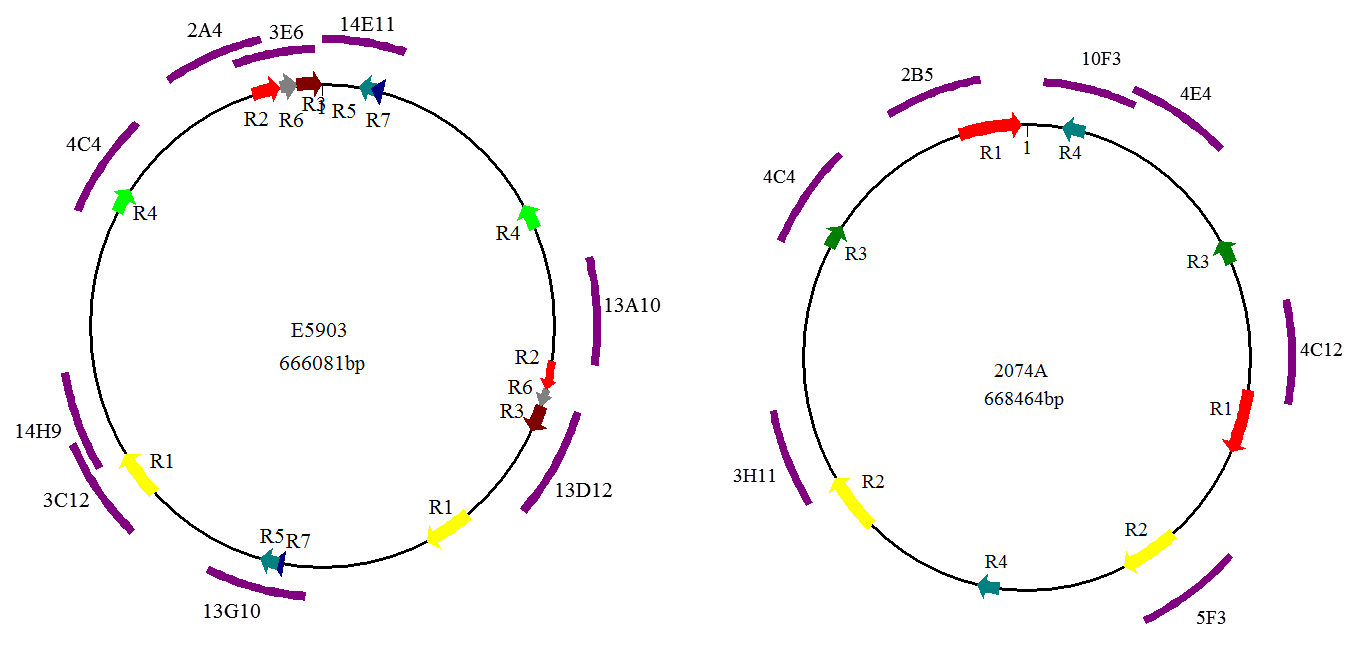


**Figure S1.** The end sequencing positive clones in E5903 and 2074A. The different color arrows represent different big repeats in two mitogenomes, and purple line represent different positive clones.
