# Supplementary figures and images for: The comparison of four mitochondrial genomes reveals cytoplasmic male sterility candidate genes in cotton
Source: BMC Genomics. 2018 Oct 26;19:775. doi: 10.1186/s12864-018-5122-y (PMC6204043; doi:10.1186/s12864-018-5122-y)

**Additional file 2:**


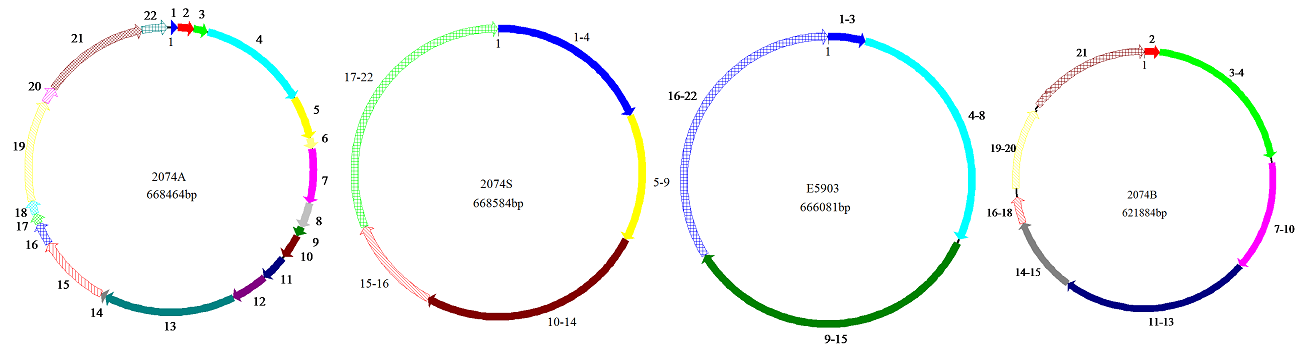


**Figure** **S2.** The syntenic regions in four mitochondrial genomes.

Supplement: Supplementary file 2 — Table S2A. The verification about breaking point of scaffolds between 2074A and 2074B. (DOCX 15 kb) [file 12864_2018_5122_MOESM2_ESM.docx]
