# Supplementary material for: The comparison of four mitochondrial genomes reveals cytoplasmic male sterility candidate genes in cotton
Source: BMC Genomics. 2018 Oct 26;19:775. doi: 10.1186/s12864-018-5122-y (PMC6204043; doi:10.1186/s12864-018-5122-y)
Supplement: Supplementary file 3 — Table S2B. The verification about breaking point of large repeats between 2074A and 2074B. (DOCX 15 kb) [file 12864_2018_5122_MOESM3_ESM.docx]

**Additional file 3:**

**Figure S3.** Differential expression of CMS candidate ORFs in 2074A, 2074B and F_1_-A. Log2 transformations of the expression fold changes (2074B/2074A and F1-A/2074A) are represented by bars. Y axis denotes the levels of transformed expression fold changes.
