# Supplementary material for: The comparison of four mitochondrial genomes reveals cytoplasmic male sterility candidate genes in cotton
Source: BMC Genomics. 2018 Oct 26;19:775. doi: 10.1186/s12864-018-5122-y (PMC6204043; doi:10.1186/s12864-018-5122-y)
Supplement: Supplementary file 4 — Table S3. The chloroplast-derived sequences (> 70 bp) found in four mitogenomes. (DOCX 19 kb) [file 12864_2018_5122_MOESM4_ESM.docx]

**Additional file 4:Table S1.** Summary of the four mitogenomes sequencing and assembly

|  | 2074A | 2074S | 2074B* | E5903 |
| --- | --- | --- | --- | --- |
| Total reads length (bp) | 4,305,700 | 1,270,225 | 3,884,873 | 1,223,368 |
| Scaffold N50 value of assembly (bp) | 38,052 | 40,063 | 693 | 39,999 |
| Number of contigs and scaffolds | 45 | 93 | 5000 | 164 |
| Maximum scaffold length (bp) | 122,783 | 120,883 | 35,878 | 120,785 |
| Number of closed gaps within scaffolds | 11 | 68 |  | 29 |
| Total length of genome (bp) | 668,464 | 668,584 | 621,884 | 666,081 |

Note. – * 2074B was sequenced by 454 sequencing technique.
