# Supplementary material for: The comparison of four mitochondrial genomes reveals cytoplasmic male sterility candidate genes in cotton
Source: BMC Genomics. 2018 Oct 26;19:775. doi: 10.1186/s12864-018-5122-y (PMC6204043; doi:10.1186/s12864-018-5122-y)
Supplement: Supplementary file 5 — Table S4. List of multi-copy genes in cotton mtDNA. (DOCX 16 kb) [file 12864_2018_5122_MOESM5_ESM.docx]

**Additional file 5:**

**Table S2A.** The verification about breaking point of scaffolds between 2074A and 2074B

| No. | ID(%) | Length(bp) | 2074B-start | 2074B-end | 2074A-start | 2074A-end | Primer(bp) | Primer-start | Primer-end |
| --- | --- | --- | --- | --- | --- | --- | --- | --- | --- |
| 1 | 99.92 | 1270 | 129821 | 131090 | 117848 | 116579 | z5(2250) | 129154 | 131404 |
| 2 | 99.69 | 11127 | 224366 | 235484 | 23285 | 12161 | 3r(2499) | 224159 | 226655 |
| 3 | 99.76 | 818 | 325922 | 326739 | 549493 | 548676 | 5-2(1142) | 325119 | 326212 |
| 4 | 99.67 | 1210 | 401887 | 403096 | 473475 | 472266 | z37(1757) | 401739 | 403496 |
| 5 | 99.93 | 5551 | 422158 | 427707 | 453150 | 447600 | bc7(3750) | 419406 | 423156 |
| 6 | 99.8 | 9091 | 427703 | 436791 | 447646 | 438558 | p1-2(2083) | 426123 | 428205 |
| 7 | 99.71 | 17646 | 436781 | 454412 | 438457 | 420819 | z2b(3509) | 453651 | 457145 |
| 8 | 99.9 | 15612 | 466926 | 482535 | 408264 | 392653 | S1-4(917) | 466409 | 467291 |
| 9 | 99.6 | 999 | 520918 | 521916 | 354045 | 353047 | b12(2002) | 520120 | 522104 |
| 10 | 99.66 | 14696 | 522112 | 536793 | 353046 | 338353 | D1(3500) | 521210 | 524709 |
| 11 | 99.75 | 12294 | 536831 | 549121 | 338319 | 326033 | 1-25(1537) | 536276 | 537744 |
| 12 | 99.78 | 3126 | 549165 | 552290 | 325989 | 322867 | 1-20(925) | 549014 | 549788 |
| 13 | 99.81 | 59647 | 552339 | 611952 | 322818 | 263185 | 1-18(877) | 554165 | 555006 |
| 14 | 99.76 | 7170 | 613573 | 620735 | 261529 | 254360 | z2(3509) | 611051 | 614525 |
