# Supplementary material for: The comparison of four mitochondrial genomes reveals cytoplasmic male sterility candidate genes in cotton
Source: BMC Genomics. 2018 Oct 26;19:775. doi: 10.1186/s12864-018-5122-y (PMC6204043; doi:10.1186/s12864-018-5122-y)
Supplement: Supplementary file 6 — Table S5. Nucleotide differences relative to the 2074B mitogenome. (DOCX 14 kb) [file 12864_2018_5122_MOESM6_ESM.docx]

**Additional file 6:**

**Table S2B.** The verification about breaking point of large repeats between 2074A and 2074B

| Repeat(start to end) | Nodal | Primer | PCR range | Primer | Nodal | The breakpoint of PCR sequence |
| --- | --- | --- | --- | --- | --- | --- |
| AR2(258403-286067) | 258403 | YZ2 | 257198-258236 | S1-5(1600) | 258403-259246 | 843-1 |
|  | 286067 | Prps3 | 285479-286376 |  |  |  |
|  |  | BC14 | 257714-259457 |  |  |  |
| AR2(415836-443499) | 415836 | S1-5 | 415221-416679 | BC14(1744) | 415836-416890 | 690-1744 |
|  | 443499 | YS18 | 443316-444978 |  |  |  |
| AR3(111514-122708) | 111514 | Z7 | 110612-113153 | Z18(1882) | 111514-112511 | 998-1 |
|  | 122708 | z5 | 121723-123738 | a9(3187) | 121492-122708 | 3187-1970 |
| AR3(553630-564820) | 553630 | a9 | 551656-554846 | z5(2016) | 553630-554615 | 1033-2016 |
|  | 564820 | z18 | 563782-565733 | z7(2542) | 563181-564820 | 1-1640 |
| AR4(17295-27926) | 17295 | YS2 | 16199-19470 | D1(3935) | 20247-17295 | 1-2943 |
|  | 27926 | E6(2534) | 25604-27926 | 2534-202 |  |  |
| AR4(347561-358192) | 347561 | E6 | 346976-349507 |  |  |  |
|  | 358192 | a12 | 358284-360267 | YS2(3272) | 356027-358192 | 3272-1107 |
|  | D1 | 355250-359184 |  |  |  |  |
| AR1(182207-211483) | 182207 | YS19 | 181243-183060 |  |  |  |
|  | 211483 |  |  |  |  |  |
| AR1(636366-665642) | 636366 | YS19(2831) | 636366-638230 | 1876-6 |  |  |
|  | 665642 |  |  |  |  |  |
