# Supplementary material for: The comparison of four mitochondrial genomes reveals cytoplasmic male sterility candidate genes in cotton
Source: BMC Genomics. 2018 Oct 26;19:775. doi: 10.1186/s12864-018-5122-y (PMC6204043; doi:10.1186/s12864-018-5122-y)
Supplement: Supplementary file 7 — Figure S1. The end sequencing positive clones in E5903 and 2074A. (DOCX 63 kb) [file 12864_2018_5122_MOESM7_ESM.docx]

**Additional file 7:**

**Table S3.** The chloroplast-derived sequences (> 70 bp) found in four mitogenomes

| NO. | 2074A | | | 2074S | | | 2074B | | | E5903 | | | tRNA^c^ |
| --- | --- | --- | --- | --- | --- | --- | --- | --- | --- | --- | --- | --- | --- |
|  | Length^a^ | start^b^ | End | Length(bp) | Start | End | Length(bp) | start | end | Length(bp) | start | end |  |
| 1 | 2203 | 382227 | 384407 | 2205 | 382277 | 384461 | 2205 | 371438 | 373622 | 995 | 385463 | 386457 |  |
|  |  |  |  |  |  |  |  |  |  | 774 | 386457 | 387230 |  |
| 2 | 1152 | 200071 | 201222 | 1152 | 200061 | 201212 | 1152 | 190819 | 191970 | 1152 | 200020 | 201169 |  |
| 3 ^df^ | 1152^df^ | 654230 | 655381 | 1152 ^df^ | 654349 | 655500 |  |  |  | 1152 ^df^ | 651846 | 652997 |  |
| 4 | 454 | 65316 | 65769 | 454 | 65317 | 65770 | 454 | 60144 | 60597 | 454 | 65323 | 65776 |  |
| 5 | 259 | 57393 | 57649 | 259 | 57394 | 57650 | 259 | 52226 | 52482 | 259 | 57400 | 57656 |  |
| 6 | 248^d^ | 188804 | 189051 | 248^d^ | 188794 | 189041 | 248^d^ | 179554 | 179801 | 248^d^ | 188824 | 189071 | *trnfM*(CAU) |
| 7 | 248 | 642963 | 643210 | 248 | 643082 | 643329 | 248 | 618246 | 618493 | 248 | 640579 | 640826 |  |
| 8 | 223 | 365678 | 365900 | 223 | 365728 | 365950 | 223 | 354894 | 355116 | 223 | 368914 | 369136 |  |
| 9 | 203 | 510779 | 510981 | 203 | 510895 | 511097 | 202 | 486066 | 486267 | 203 | 508679 | 508881 |  |
| 10 | 152 | 42069 | 42219 | 152 | 42070 | 42220 | 152 | 36900 | 37050 | 152 | 42077 | 42227 |  |
| 11 | 143 | 66743 | 66885 | 143 | 66744 | 66886 | 143 | 61573 | 61715 | 143 | 66750 | 66892 | *trnW*(CCA) |
| 12 | 138 | 57264 | 57399 | 138 | 57265 | 57400 | 138 | 52097 | 52232 | 138 | 57271 | 57406 |  |
| 13 | 119 | 613718 | 613836 | 119 | 613835 | 613953 | 119 | 588976 | 589094 | 119 | 611718 | 611836 |  |
| 14 | 118 | 614501 | 614617 | 118 | 614618 | 614734 | 118 | 589759 | 589875 | 118 | 612501 | 612617 |  |
| 15 | 101 | 65899 | 65999 | 101 | 65900 | 66000 | 101 | 60725 | 60825 | 101 | 65906 | 66006 |  |
| 16 | 98 | 379145 | 379242 | 98 | 379195 | 379292 | 98 | 368356 | 368453 | 98 | 382381 | 382478 |  |
| 17 | 95 | 391816 | 391910 | 95 | 391910 | 392004 | 95 | 381021 | 381115 | 95 | 394650 | 394744 | *trnW*(CCA) |
| 18 | 90 | 188347 | 188436 | 90 | 188337 | 188426 | 90 | 179097 | 179186 | 90 | 188367 | 188456 |  |
| 19^f^ | 90^f^ | 642506 | 642595 | 90 ^f^ | 642625 | 642714 | 87 | 589621 | 589707 | 90 ^f^ | 640122 | 640211 |  |
| 20 | 87 | 614363 | 614449 | 87 | 614480 | 614566 | 86 | 588413 | 588498 | 87 | 612363 | 612449 |  |
| 21 | 86 | 613155 | 613240 | 86 | 613272 | 613357 | 85^d^ | 178981 | 179065 | 86 | 611155 | 611240 |  |
| 22 | 85^d^ | 188231 | 188315 | 85^d^ | 188221 | 188305 | 85 | 617671 | 617755 | 85^d^ | 188251 | 188335 |  |
| 23 | 85 | 642390 | 642474 | 85 | 642509 | 642593 | 84 | 61355 | 61438 | 85 | 640006 | 640090 |  |
| 24 | 84 | 66525 | 66608 | 84 | 66526 | 66609 | 84 | 543334 | 543417 | 84 | 66532 | 66615 |  |
| 25 | 84 | 568070 | 568153 | 84 | 568186 | 568269 | 82^g^ | 617795 | 617876 | 84 | 565984 | 566067 | *trnD*(GUC) |
| 26 | 80 | 219841 | 219920 | 80 | 219888 | 219967 | 80 | 210587 | 210666 | 80 | 223045 | 223124 | *trnS*(GGA) |
| 27 | 79 | 252468 | 252546 | 79 | 252515 | 252593 | 79 | 241706 | 241784 | 79 | 255672 | 255750 | *trnN*(GUU) |
| 28 | 78 | 90847 | 90924 | 78 | 90849 | 90926 | 78 | 85669 | 85746 | 78 | 90854 | 90931 | *trnfM*(CAU) |
| 29 | 76 | 46460 | 46535 | 76 | 46461 | 46536 | 76 | 41294 | 41369 | 76 | 46468 | 46543 | *trnD*(GUC) |
| 30 | 75 | 543038 | 543112 | 75 | 543154 | 543228 | 75 | 518324 | 518398 | 75 | 540938 | 541012 | *trnH*(GUG) |
| 31 | 75 | 613882 | 613956 | 75 | 613999 | 614073 | 75 | 589140 | 589214 | 75 | 611882 | 611956 |  |
| Total-L^e^ | 8260 |  |  | 8262 |  |  | 7101 |  |  | 7826 |  |  |  |

Note. – ^a^ length in bp; ^b^ the start and end sites of chloroplast-derived sequences in corresponding mitogenomes; ^c^ these chloroplast-derived sequences contain tRNAs; ^d^ these sequences have two copies; ^e^ total length; ^f^ these sequences in 2074A, 2074S and E5903; ^g^ this sequence unique in 2074B.
