# Supplementary material for: The comparison of four mitochondrial genomes reveals cytoplasmic male sterility candidate genes in cotton
Source: BMC Genomics. 2018 Oct 26;19:775. doi: 10.1186/s12864-018-5122-y (PMC6204043; doi:10.1186/s12864-018-5122-y)
Supplement: Supplementary file 8 — Figure S2. The syntenic regions in four mitochondrial genomes. (DOCX 80 kb) [file 12864_2018_5122_MOESM8_ESM.docx]

**Additional file 8:**

**Table S4.** List of multi-copy genes in cotton mtDNA

| Gene | Size  (bp) | 2074A | | 2074S | | E5903 | | 2074B | |
| --- | --- | --- | --- | --- | --- | --- | --- | --- | --- |
|  |  | from | To | From | To | from | to | From | to |
| *nad1* | 1243 | 425917 | 427159 | 426011 | 427253 | 428752 | 429994 | 415100 | 416342 |
| *nad1exon2* | 81 | 425921 | 426001 | 426015 | 426095 | 428756 | 428836 | 415104 | 415184 |
| *nad1exon3* | 192 | 426968 | 427159 | 427062 | 427253 | 429803 | 429994 | 416151 | 416342 |
| *rrn26* | 3374 | 640120 | 643493 | 640218 | 643591 | 637716 | 641089 | 615369 | 618744 |
| *rps3* | 2956 | 440544 | 443499 | 440639 | 443594 | -^a^ | - | 429719^b^ | 432575 |
| *CcmFC* | 2308 | 661099 | 663406 | 661197 | 663504 | 658695 | 661002 | - | - |
| *trnD(GUC)-cp* | 74 | 568093 | 568166 | 568188 | 568261 | 548831 | 548904 | 543325 | 543398 |
| *trnfM(CAU)-cp* | 73 | 189677 | 189749 | 189667 | 189739 | 189697 | 189769 | 180414 | 180486 |
| *trnfM(CAU)-cp* | 73 | 543061 | 543133 | 543156 | 543228 | 540941 | 541013 | 518315 | 518387 |
| *trnfM(CAU)-cp* | 73 | 643857 | 643929 | 643955 | 644027 | 641453 | 641525 | 619108 | 619180 |
| *trnP(UGG)* | 75 | 267050 | 267124 | 424577 | 424651 | 270254 | 270328 | 256271 | 256345 |
| *trnP(UGG)* | 75 | 424483 | 424557 | 267097 | 267171 | 427318 | 427392 | 413666 | 413740 |
| *trnS(GCU)* | 88 | 423754 | 423841 | 423848 | 423935 | 426589 | 426676 | 412937 | 413024 |
| *trnW(CCA)-cp* | 74 | 379166 | 379239 | 379216 | 379289 | 382476 | 382403 | 368366 | 368439 |
| *trnF(GAA)* | 74 | 424189 | 424262 | 424283 | 424356 | 427024 | 427097 | 413372 | 413445 |
| *trnM(CAU)* | 81 | 657547 | 657627 | 657645 | 657725 | 655143 | 655223 | 527955 | 528040 |

Note. – ^a^ not detected; ^b^ *rps3* was 2857 bp in 2074B.
