# Supplementary material for: The comparison of four mitochondrial genomes reveals cytoplasmic male sterility candidate genes in cotton
Source: BMC Genomics. 2018 Oct 26;19:775. doi: 10.1186/s12864-018-5122-y (PMC6204043; doi:10.1186/s12864-018-5122-y)
Supplement: Supplementary file 9 — Figure S3. Differential expression of CMS candidate ORFs in 2074A, 2074B and F1-A. Log2 transformations of the expression fold changes (2074B/2074A and F1-A/2074A) are represented by bars. Y axis denotes the levels of transformed expression fold changes. (DOCX 19 kb) [file 12864_2018_5122_MOESM9_ESM.docx]

**Additional file 9:**

**Table S5.** Nucleotide differences relative to the 2074B mitogenome.

| Total genome | | | | | | | Genes | | |
| --- | --- | --- | --- | --- | --- | --- | --- | --- | --- |
| Genome | No. of nucleotide substitutions | Nucleotide substitutions /10 kb^a^ | No. of indel | Indel /10 kb | Total length of indel (bp) | Variation percent coverage of total genome | Length (bp)^b^ | No. of nucleotide substitutions | Nucleotide substitutions /10 kb |
| 2074A | 266 | 3.98 | 219 | 3.28 | 425 | 0.064 | 71,320 | 11 | 1.54 |
| 2074S | 187 | 2.80 | 188 | 2.81 | 393 | 0.059 | 71,320 | 9 | 1.26 |
| E5903 | 228 | 3.42 | 192 | 2.88 | 428 | 0.064 | 71,320 | 10 | 1.40 |

Note. –^a^ Nucleotides in total length of the genomes; ^b^ Number of common nucleotides in single copies of all of the genes.
